# SwinTCS: A Swin Transformer Approach to Compressive Sensing with Non-Local Denoising

**DOI:** 10.3390/jimaging11050139

**Published:** 2025-04-29

**Authors:** Xiuying Li, Haoze Li, Hongwei Liao, Zhufeng Suo, Xuesong Chen, Jiameng Han

**Affiliations:** 1 Beijing Electronic Science and Technology Institute, Beijing 100071, China; lixiuying@besti.edu.cn (X.L.); 20233901@mail.besti.edu.cn (H.L.); 20233911@mail.besti.edu.cn (H.L.); 20223906@mail.best.edu.cn (X.C.); 20232924@mail.besti.edu.cn (J.H.); 2 Laboratory of Space-Air-Ground-Ocean Intergrated Network Security, School of Cyberspace Security, Hainan University, Haikou 570228, China

**Keywords:** compressive sensing, Swin Transformer, non-local means denoising, CNN, image reconstruction

## Abstract

In the era of the Internet of Things (IoT), the rapid growth of interconnected devices has intensified the demand for efficient data acquisition and processing techniques. Compressive Sensing (CS) has emerged as a promising approach for simultaneous signal acquisition and dimensionality reduction, particularly in multimedia applications. In response to the challenges presented by traditional CS reconstruction methods, such as boundary artifacts and limited robustness, we propose a novel hierarchical deep learning framework, SwinTCS, for CS-aware image reconstruction. Leveraging the Swin Transformer architecture, SwinTCS integrates a hierarchical feature representation strategy to enhance global contextual modeling while maintaining computational efficiency. Moreover, to better capture local features of images, we introduce an auxiliary convolutional neural network (CNN). Additionally, for suppressing noise and improving reconstruction quality in high-compression scenarios, we incorporate a Non-Local Means Denoising module. The experimental results on multiple public benchmark datasets indicate that SwinTCS surpasses State-of-the-Art (SOTA) methods across various evaluation metrics, thereby confirming its superior performance.

## 1. Introduction

The Internet of Things (IoT) is one of the most transformative technological trends today [1]. By connecting an extensive network of devices and sensors, IoT significantly enhances real-time data processing and intelligence, driving advancements in areas such as smart healthcare and smart homes [2]. However, the vast volume of data generated by IoT [3], particularly visual information such as images and videos, presents substantial challenges in terms of storage, processing, and transmission [4]. These challenges have increasingly become bottlenecks, hindering the further development of IoT.

First, the traditional Nyquist–Shannon sampling theorem necessitates a high sampling rate, which is often impractical for IoT sensors due to hardware limitations, particularly in low-power scenarios [5]. Additionally, the high energy consumption associated with data transmission and storage contradicts the lightweight design principles of IoT [6]. Moreover, privacy concerns are growing. Visual data frequently contain sensitive information, and large-scale transmission coupled with centralized storage could result in significant privacy breaches. These issues underscore the urgent need for efficient, low-redundancy methods for data sampling and processing.

Compressive Sensing (CS) [7] has emerged as a promising solution to these challenges. By exploiting the sparsity of signals, CS dramatically reduces the data required for sampling while still enabling high-quality reconstruction. This approach alleviates the storage and energy consumption burdens while minimizing the redundant collection of sensitive data, thereby enhancing privacy protection [8]. CS has become an essential tool across various domains, including IoT, medical imaging, and computer vision. Its applications range from single-pixel cameras [9] and magnetic resonance imaging (MRI) [10] to underwater imaging [11], remote sensing [12], astronomical image processing, and ultrasound imaging, offering critical support for a data-driven future.

Reconstruction quality and noise robustness are crucial factors in assessing the performance of compressive sensing models. Traditional methods, including greedy algorithms [13] and convex optimization [14], have been widely explored for solving sparse optimization problems. However, these approaches often fail to fully leverage the inherent characteristics of the data. Additionally, their iterative nature tends to introduce high computational complexity and impose stringent hardware requirements, limiting their practicality.

In recent years, deep learning techniques have significantly enhanced the reconstruction quality and speed of compressive sensing. For instance, block-sampling-based strategies, such as IWR [15] and BCSNet [16], aim to reduce the storage requirements of measurement matrices. Neural-network-based reconstruction methods, including ADMM-CSNet [17], NeumNet [18], ISTA-Net [19], and AMP-Net [20], improve upon traditional algorithms by leveraging deep learning features. End-to-end methods, such as DR2-Net [21], ReconNet [22], and CSNet [23], employ Convolutional Neural Networks (CNNs) to achieve efficient image reconstruction, making them well suited for lightweight communication scenarios. However, despite their strength in capturing local image features, these CNN-based models exhibit limitations in effectively modeling global information.

The advent of Transformer models has brought significant breakthroughs in reconstruction algorithms. Models like OCTUF [24], CSformer [25], and TransCS [26] exploit the strength of Transformers in capturing global features, substantially improving reconstruction quality and computational speed. Nevertheless, the high computational complexity and memory overhead inherent in Transformer models pose notable challenges for practical hardware deployment.

To address the issues in former compressive sensing image reconstruction methods, we propose a dual-path compressive sensing model that integrates the Swin Transformer and CNN. The model integrates an enhanced attention mechanism and advanced denoising strategies to improve reconstruction quality and robustness. Inspired by the shifted window attention mechanism in the Swin Transformer, the model effectively captures both local and global features. Additionally, it incorporates a convolutional neural network (CNN) module, which excels at efficiently extracting local image details. By leveraging the Transformer module’s capability to capture global features and the CNN module’s proficiency in extracting local details, the model effectively integrates both global and local information. These features are subsequently fused through a comprehensive integration mechanism, enhancing the coherence and interaction between the global context and local details. This design not only significantly reduces artifact generation but also improves image reconstruction quality, optimizing metrics such as PSNR [27] and SSIM [28] while maintaining computational efficiency.

The specific contributions of this paper are as follows:

- We propose a novel deep compressive sensing framework named SwinTCS, which integrates the shifted window Transformer (Swin Transformer) and convolutional neural network (CNN) to enhance the quality and efficiency of image reconstruction. The Swin Transformer, with its shifted window attention mechanism, effectively eliminates boundary artifacts inherent in block-based compressive sensing models while significantly reducing computational complexity. Meanwhile, the CNN component strengthens local feature extraction, further optimizing the overall performance of the model.
- We design an advanced noise suppression module within the SwinTCS framework, utilizing the Non-Local Feature Means (NLM) algorithm to enhance model robustness. This module leverages the global similarity of image features to effectively mitigate the impact of complex noise, improving the reconstruction process’s adaptability and stability under diverse noise conditions.
- We introduced an Attention Fusion module in SwinTCS, which integrates global features captured by the Transformer with local features extracted by the CNN. This module further enhances the interaction and consistency between global and local information, significantly improving image reconstruction quality and detail recovery.

This paper is organized as follows. In Section 2, we present the related works, mainly including mathematical modeling methods for CS, DCS (Deep Compressive Sensing), Swin Transformer, and NLM. Section 3 presents the proposed SwinTCS framework, including the detailed modeling process of the signal sampling module and the reconstruction module, as well as a discussion of the comparison with other models. In Section 4, we verify the validity of the proposed SwinTCS model by presenting the experimental results of SwinTCS and other competing models. Finally, a summary of the article and directions for future work are given in Section 5.

## 2. Related Works

This section gives a brief presentation of related works, including Deep Compressive Sensing, Swin Transformer, and Non-Local Means Denoising (NLM).

### 2.1. Deep Compressive Sensing

Compressive Sensing (CS) leverages the sparsity of signals and incoherent sampling to enable complete signal reconstruction with a sampling rate lower than the Nyquist rate. Suppose a signal $x\in R^{N}$ needs to be sampled, and the signal is sparse in a certain domain. CS theory ensures that the signal can be reconstructed using a small number of linear projections:

(1) y=Φx

where Φ is the observation matrix with dimensions M×N and *K* nonzero elements (K≪N). Since M<N, this equation represents an underdetermined linear system. Generally, computing *x* from the known *y* is challenging because there exist infinitely many solutions $x^{\prime }$ such that $y=\Phi x^{\prime }$. However, under the assumption that *x* is sparse, complete reconstruction becomes feasible.

CS recovers the original signal by solving an optimization problem, aiming to minimize the error between the observation *y* and the reconstructed signal $\Phi^{-1}y$ while promoting sparsity in the reconstructed signal. Although using the L0 norm is the optimal strategy, its optimization is NP-hard. In contrast, the L1 norm serves as a convex approximation to the L0 norm and is widely used in practice. Consequently, the optimization problem can be reformulated with the L1 norm as a sparsity regularization term, as follows:

(2) $${\min||s||}_{1}\;\mathrm{subject}\;\mathrm{to}\;\Phi \Psi s=y$$

where ${\left||s|\right|}_{1}$ represents the L1 norm of *s*. Solving this optimization problem provides the sparsest solution *s*, which allows the original signal to be reconstructed as x=Ψs, with Ψ representing the sparse group.

Deep Compressive Sensing (DCS) leverages neural networks to learn the complex mapping between measurements and the original signal. This approach enhances the speed and accuracy of the reconstruction process, optimizing overall image sampling and reconstruction performance. DCS typically addresses the problem by minimizing:

(3) $$\min||x-g_{\Phi }\left(y\right){\left|\right|}_{2}$$

where *x* is the original signal, *y* is the observation (i.e., the network input), and $g_{\Phi }\left(\cdot \right)$ is the inverse transformation function defined by the network parameters Φ. As deep learning evolves, various DCS algorithms have been proposed, which can be categorized into two main types:

#### 2.1.1. Category I: Traditional Model-Driven Approaches

Combining traditional CS algorithms with deep learning through iterative computation helps maintain stability while improving reconstruction quality and speed. For example, ISTA-Net replaces the sparsity constraint in the linear transform domain of traditional ISTA with one in the nonlinear transform domain. AMP-Net extends the Approximate Message Passing (AMP) algorithm to deep learning. TransCS introduces a Transformer backbone based on ISTA, modeling global dependencies between image sub-blocks and performing iterative gradient descent and soft thresholding. DRCAMP-Net [29] combines AMP with extended residual convolution to remove block artifacts and expand the receptive field. LTwIST [30] uses a two-step IST algorithm with a trainable induction matrix to learn image structure and solves the proximal mapping problem via a U-block architecture.

#### 2.1.2. Category II: Data-Driven Deep Learning Approaches

Deep learning models, such as Convolutional Neural Networks (CNNs), are increasingly used for compression sensing (CS) reconstruction. These models focus on capturing local features by stacking convolutional layers. For example, DR2-Net leverages linear mapping and residual networks for image reconstruction, while ReconNet directly reconstructs original images through convolutional layers. DPA-Net enhances reconstruction quality by preserving texture details, and CSNet enables fast end-to-end image reconstruction using residual networks. CSformer combines the spatial information from CNNs with the global context provided by Transformers to improve feature learning. MSCRLNet [31] incorporates multi-scale residual networks to enhance attention to shallow features, whereas D3C2-Net [32] integrates prior knowledge from image and convolutional coding domains to efficiently transmit adaptive convolution features. While these methods significantly improve reconstruction quality, they also encounter challenges related to computational complexity and memory requirements, particularly when Transformer-based models are involved.

### 2.2. Swin Transformer

Traditional Transformer architectures, despite their powerful global modeling capabilities, suffer from high computational complexity when processing high-resolution images. This limitation makes them less practical for large-scale tasks such as Compressive Sensing (CS) image reconstruction. To address this, Swin Transformer [33] introduces a more efficient architecture based on Shifted Window Self-Attention, offering a balance between local and global feature modeling while significantly reducing computation.

Unlike the Vision Transformer (ViT) [34], which performs global self-attention over fixed-size image patches and incurs quadratic computational cost, Swin Transformer divides images into non-overlapping patches and computes self-attention within local windows. To capture long-range dependencies, it introduces a shifted window mechanism that allows cross-window interaction without sacrificing efficiency. This design makes Swin Transformer particularly suitable for CS image reconstruction tasks that require both contextual understanding and computational scalability.

**Figure 1:**
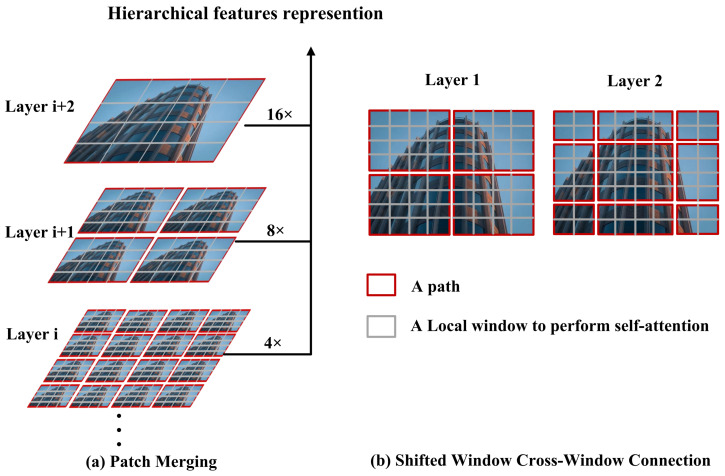
Illustration of shifted windows and patch merging in Swin Transformer.

Figure 1 illustrates the shifted window and patch merging operations. The input image *I* is first partitioned into patches and linearly embedded into feature vectors:

(4) z=PatchEmbed(I)

Within each Transformer block, attention is computed locally within windows:

(5) $$\mathrm{Attention}\left(Q,K,V\right)=\mathrm{softmax}\left(\frac{QK^{T}}{\sqrt{d_{k}}}\right)V$$

where *Q*, *K*, and *V* are the Query, Key, and Value matrices, and $d_{k}$ is the dimensionality of the Key.

To enhance the receptive field, the Swin Transformer alternates between regular and shifted window configurations across layers. This creates overlapping attention regions and promotes information flow between local regions, effectively modeling long-range dependencies without introducing global self-attention.

In addition, Swin Transformer adopts a hierarchical architecture, where spatial resolution is gradually reduced through Patch Merging:

(6) $$z_{\mathrm{merged}}=\mathrm{PatchMerge}\left(z\right)$$

This operation reduces the spatial dimensions while increasing channel depth, enabling efficient multi-scale feature representation—an essential property for reconstructing high-quality images from compressed measurements.

The Swin Transformer has demonstrated state-of-the-art performance in image classification, detection, segmentation [35], and, more recently, in image restoration and CS. In CS image reconstruction, it is often integrated with Convolutional Neural Networks (CNNs) to extract fine-grained local features. These hybrid architectures combine Swin Transformer’s global context modeling and CNNs’ local detail preservation, leading to superior performance in terms of PSNR and SSIM, especially under high compression ratios. This makes Swin Transformer-based approaches both effective and practical for real-world CS applications.

### 2.3. Non-Local Means Denoising

Non-Local Means (NLM) denoising [36] is a classical image denoising method that suppresses noise by leveraging the global correlations of features. This method adjusts pixel values based on the similarity weights between a pixel and other distant pixels, effectively capturing non-local dependencies. Initially proposed for image denoising, NLM is notable for its ability to restore image details under strong noise conditions, addressing the challenge of capturing long-range pixel dependencies that traditional local methods struggle with. As research has progressed, this method has been applied to a broader range of fields, including medical imaging [37] and remote sensing image analysis [38].

In recent years, the concept of NLM has been introduced into deep learning, forming Non-Local Neural Networks (NLNNs). These models compute similarities and aggregate features through global attention mechanisms, significantly enhancing feature modeling capabilities [39]. The similarity computation and feature aggregation can be expressed as follows:

Attention Mechanism:

(7) $$\mathrm{Attention}\left(i,j\right)=\mathrm{softmax}\left(\theta {\left(x_{i}\right)}^{⊤}\xi \left(x_{j}\right)\right)$$

where θ, ξ, and ν are mapping functions for dimensionality reduction, and *i* and *j* denote pixel indices.

Output Computation:

(8) $$\mathrm{Output}\left(i\right)=\sum_{j}\mathrm{Attention}\left(i,j\right)\nu \left(x_{j}\right)$$

By leveraging these mechanisms, NLM denoising can significantly improve the robustness of compressive sensing models and the overall coherence of image restoration. Moreover, compared to traditional methods such as Approximate Message Passing (AMP) and Block-Matching and 3D Filtering (BM3D) [40], NLM does not rely on explicit signal priors but instead performs denoising by dynamically computing pixel similarities. This provides greater flexibility and enhanced application potential.

## 3. Proposed Method

**Figure 2:**
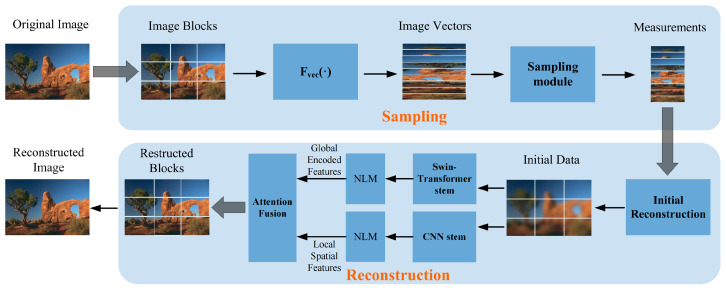
Overall architecture of SwinTCS.

In this section, we will introduce the proposed network model, whose architecture is shown in Figure 2. As can be seen from the figure, the model is mainly divided into two modules: a learnable sampling module and a reconstruction module. The reconstruction module first performs an initial reconstruction, followed by two parallel processing paths: one based on the Swin Transformer to capture global features and the other using the CNN to extract local features. The outputs from these two paths are then processed through a Non-Local Denoising Layer (NLM) to further refine the feature maps by reducing noise and enhancing key image features. This denoised representation is then fused through a fusion attention-based aggregation mechanism to generate the final reconstructed result. Additionally, auxiliary convolutional layers are introduced between these two main modules to further enhance reconstruction performance.

### 3.1. Sampling Module

To achieve better image reconstruction performance, the sampling module employs a data-driven trainable sensing matrix. Specifically, the input image *x* is first divided into C×B×B non-overlapping blocks, where *C* represents the number of image channels, and *B* denotes the block size. Let the sensing matrix be $A\in R^{M\times N}$, where $M=\tau \cdot B^{2}$, $N=B^{2}$, and the sampling rate is defined as $\tau =\frac{M}{N}$ (0≤τ≤1). For example, when the sampling rate τ=0.1, C=1, and B=32, the size of the sensing matrix is 102×1024 (102 rows and 1024 columns).

The sampling module uses a blocking function $F_{B}\left(\cdot \right)$ to divide the original image into B×B non-overlapping blocks and then applies a flattening function $F_{\mathrm{vec}}\left(\cdot \right)$ to transform the blocks into vectors. Subsequently, the elements of the sensing matrix *A*, which are initialized following a normal distribution, are automatically optimized through joint training with the reconstruction module. This process learns the distribution from training images and converges to a form resembling a Gaussian distribution. Therefore, the sampling process can be formulated as

(9) $$y=S\left(x,A\right)=A\cdot F_{\mathrm{vec}}\left(F_{B}\left(x\right)\right)$$

where S(·,A) represents the sampling process.

Through joint training with the reconstruction module, we obtain a learned floating-point sensing matrix *A*, which enhances the reconstruction performance. Similar to CSNet, constraints can be applied to the elements of *A* to enforce a binary pattern {0,1}, resulting in a data-driven binary sensing matrix. Unlike floating-point sensing matrices, the learned binary sensing matrix is more efficient in terms of hardware implementation and storage requirements.

### 3.2. Reconstruction Module

The reconstruction part of the model mainly consists of two components: the Initial Reconstruction Module and the Hybrid Reconstruction Module. In addition, Non-Local Means Denoising is introduced after the initial reconstruction to enhance the robustness of the reconstruction performance and make the reconstructed images more natural.

#### 3.2.1. Initial Reconstruction

During the initial reconstruction process, we introduce a learnable initial reconstruction matrix $\tilde{A}$ to perform a preliminary restoration of the image. The matrix $\tilde{A}$ is initialized as the transpose of the sampling matrix *A* and is automatically trained through backpropagation. For a sampling rate $\tau =\frac{M}{N}$, the measurements *y* (obtained from the sampling module S(x,A)) consist of $\left[MB^{2}/N\right]$ rows. Therefore, the initial reconstruction process can be expressed as

(10) $$x_{\mathrm{init}}=L\left(y,\tilde{A}\right)=\tilde{A}\cdot y=\tilde{A}\cdot S\left(x,A\right)$$

where $L(\cdot ,\tilde{A})$ represents the initial reconstruction submodule, and $x_{\mathrm{init}}$ denotes the initial restoration result.

However, relying solely on the initial reconstruction submodule $L(\cdot ,\tilde{A})$ alone is insufficient as its limited capacity hinders the ability to fully preserve the image information in the reconstructed output. Additionally, this submodule suffers from blocking artifacts, where artifacts may appear at the boundaries of image blocks after reconstruction. To improve image reconstruction accuracy and mitigate blocking artifacts, we design a Hybrid Reconstruction Submodule, which takes $x_{\mathrm{init}}$ as its input.

#### 3.2.2. Hybrid Reconstruction

**Figure 3:**
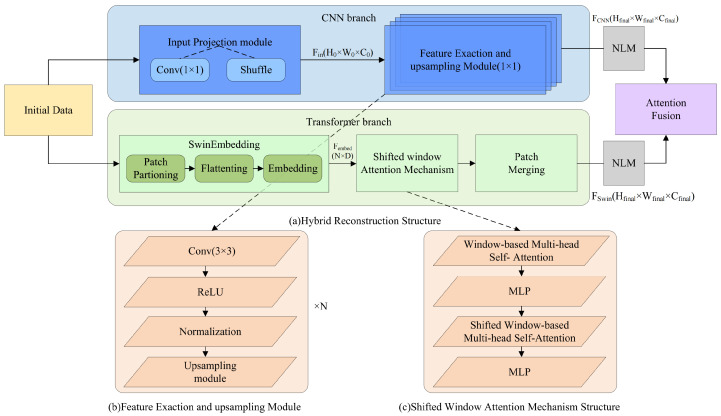
SwinTCS hybrid reconstruction module with CNN and Transformer branches for improved reconstruction.

The proposed hybrid reconstruction model comprises two parallel branches to effectively capture both local and global image features. The first branch employs a CNN-based structure to extract fine-grained local details, while the second branch leverages the Swin Transformer to model long-range dependencies and global contextual information. The outputs from these two branches are subsequently processed through a Non-Local Denoising Layer (NLM) to further refine the feature maps, reducing noise and enhancing key image structures. After denoising, the features are integrated through a fusion attention-based aggregation mechanism module to generate the final reconstructed image. This dual-branch architecture, combined with the NLM, ensures a comprehensive representation of image features, thereby significantly improving the reconstruction accuracy and visual quality. The overall framework of the hybrid reconstruction model is illustrated in Figure 3.

a CNN branch

In the hybrid reconstruction module, the CNN branch is primarily responsible for extracting local detail features. The initial reconstruction result $x_{\mathrm{init}}$ passes through this branch to generate fine-grained local feature representations $F_{\mathrm{CNN}}$, which supports the final image reconstruction.

Specifically, the input of the CNN branch is the initial reconstruction result $x_{\mathrm{init}}\in R^{H\times W\times C}$. This input is first processed by an input projection module, consisting of several 1×1 convolution layers followed by a pixel shuffle operation. This module generates the initial feature representation $F_{\mathrm{in}}\in R^{H_{0}\times W_{0}\times C_{0}}$, typically setting $H_{0}=W_{0}=8$.

Next, $F_{\mathrm{in}}$ is fed into the CNN backbone network for local feature extraction. The CNN backbone consists of multiple convolution blocks, where each block includes two convolutional layers, followed by a Leaky ReLU activation function and a batch normalization layer. Each convolutional layer has a kernel size of 3×3 with a padding size of 1, and the output channels remain consistent with the input channels. The feature representations maintain the same resolution and channel size after passing through each convolution block.

To gradually upscale the feature resolution, an upsampling module is added between convolution blocks. This module first uses bicubic interpolation to double the resolution of the feature, followed by a 1×1 convolution layer to halve the channel dimensions. The output feature of the upsampling module can be represented as $F_{\mathrm{up}}$.

After multi-level convolutions and upsampling, the final local feature representation is denoted as $F_{\mathrm{CNN}}\in R^{H_{\mathrm{final}}\times W_{\mathrm{final}}\times C_{\mathrm{final}}}$, whose resolution matches that of the initial input image.

b Swin Transformer branch

In the hybrid reconstruction module, the Transformer branch is designed to capture global image features and long-range dependencies. Unlike conventional CNN-based methods, which focus on local feature extraction, this branch leverages the Swin Transformer as the backbone network. By incorporating shifted window-based self-attention and hierarchical feature representations, the Transformer branch effectively models global contextual information, mitigating the limitations of previous Transformer architectures in compressed sensing image reconstruction. The main components of this branch include Swin Embedding, Shifted window Attention Mechanism, and Patch Merging.

The Transformer branch takes the initial reconstruction result $x_{\mathrm{init}}\in R^{H\times W\times C}$ as input. Before passing through the Swin Transformer backbone, the input is processed by an embedding module that transforms pixel-level representations into a high-dimensional feature space. Specifically, the input image is divided into non-overlapping patches of size P×P, resulting in a sequence of flattened patch tokens. Each patch is then mapped to a *D*-dimensional feature space via a linear embedding layer, generating embedded patch tokens $F_{\mathrm{embed}}$. The key mathematical formulations involved in these processes are summarized in Algorithm 1.

The embedded features $F_{\mathrm{embed}}$ are subsequently passed through a series of Swin Transformer blocks, each consisting of multi-head self-attention (WMSA and SWMSA) and a multilayer perceptron (MLP) for feature transformation. In the WMSA module, the input features are partitioned into non-overlapping windows of size M×M, and standard multi-head self-attention is applied within each window. After applying the attention mechanism, the features are processed by an MLP for nonlinear transformation.

To capture cross-window dependencies, the shifted window mechanism (SWMSA) is introduced in alternating Swin Transformer blocks. The window partitioning is shifted by ⌊M/2⌋ pixels in both horizontal and vertical directions. This ensures overlap between neighboring windows, enabling information exchange across windows. For shifted windows, zero-padding is applied at boundaries to maintain consistent dimensions. The SWMSA features are similarly processed using self-attention and an MLP.

**Algorithm 1** Formulations in the Transformer Branch.**Operation**                              Mathematical Expression**Input**                                      Image feature map $x_{\mathrm{init}}$**Output**                                   Processed feature map $x_{\mathrm{out}}$1: Patch Reshaping                $x_{\mathrm{patch}}=\mathrm{Reshape}\left(x_{\mathrm{init}}\right),\;x_{\mathrm{patch}}\in R^{\frac{H}{P}\;\times \;\frac{W}{P}\;\times \;\left(P^{2}\cdot C\right)}$2: Patch Embedding              $F_{\mathrm{embed}}=\mathrm{Linear}\left(x_{\mathrm{patch}}\right),\;F_{\mathrm{embed}}\in R^{N\;\times \;D}$3: N Calculation                    $N=\frac{H}{P}\cdot \frac{W}{P}$4: QKV Calculation               $Q,K,V=F_{\mathrm{embed}},\;Q,K,V\in R^{M^{2}\;\times \;d_{k}}$5: $d_{k}$ Calculation                    $d_{k}=\frac{D}{h}$6: Attention Calculation       $F_{\mathrm{attn}}=\mathrm{Softmax}\left(\frac{QK^{⊤}}{\sqrt{d_{k}}}\right)V$7: WMSA Output                  $F_{\mathrm{WMSA}}=\mathrm{MLP}\left(F_{\mathrm{attn}}\right)$8: SWMSA Output                $F_{\mathrm{SWMSA}}=\mathrm{MLP}\left(F_{\mathrm{attn}}^{\prime }\right)$9: Residual Connection        $F_{\mathrm{block}}=F_{\mathrm{input}}+F_{\mathrm{WMSA}/\mathrm{SWMSA}}$10: Patch Merging                 $F_{\mathrm{merge}}=\mathrm{Linear}\left(\mathrm{Concat}\left(F_{{\mathrm{patch}}_{1}},\ldots ,F_{{\mathrm{patch}}_{k}}\right)\right)$                                                $F_{\mathrm{merge}}\in R^{\frac{H}{2}\;\times \;\frac{W}{2}\;\times \;2C}$11: **Output**                             $x_{\mathrm{out}}=F_{\mathrm{merge}}$

To construct a hierarchical feature representation, a patch merging module is employed at the end of certain Swin Transformer stages. This module reduces the spatial resolution of feature maps while increasing their channel dimensions, effectively summarizing global features at different scales. In the patch merging operation, adjacent non-overlapping patches are concatenated and passed through a linear layer.

Finally, the Transformer branch outputs the global feature representations $F_{\mathrm{Swin}}\in R^{H_{\mathrm{final}}\;\times \;W_{\mathrm{final}}\;\times \;C_{\mathrm{final}}}$, which are then fused with the local features $F_{\mathrm{CNN}}$ from the CNN branch in the feature fusion module. This integration leverages both global and local information, producing a high-quality final image reconstruction.

##### Non-Local Denoising Layer

After the feature representations from both the CNN and Transformer branches have been extracted, a Non-Local Denoising Layer (NLM) is applied to further refine the feature maps by reducing noise and enhancing the key features. This layer utilizes non-local attention mechanisms to capture long-range dependencies between distant pixels, allowing it to effectively suppress noise while preserving important image structures.

The NLM processes the output feature maps from the previous stages, which are first passed through a series of convolution layers. These layers reduce the channel dimensions, followed by batch normalization to stabilize the learning process. The resulting feature maps are then refined through a non-local attention mechanism, which computes attention maps by comparing the features across the entire spatial domain. The attention maps are used to weight the feature maps, allowing the model to focus on relevant regions of the image.

Additionally, a Squeeze-and-Excitation (SE) block is incorporated to adjust channel-wise attention, ensuring that important channels receive more emphasis while less informative channels are suppressed. The final output is a denoised feature map that is used for the final image reconstruction.

By removing noise and enhancing the features, the NLM plays a critical role in improving the visual quality and accuracy of the final reconstruction.

##### Feature Fusion Module

Following the refinement of feature representations through the Non-Local Denoising Layer, the feature fusion module integrates the outputs of the CNN and Transformer branches to produce the final reconstructed image. By combining local detail features and global contextual features, this module ensures a comprehensive representation of image characteristics.

The feature fusion module takes the features from the CNN and Transformer branches as input, which are aligned in terms of spatial resolution and channel dimensions. These input features are first concatenated along the channel dimension and then passed through a series of convolutional layers for feature refinement and integration. Each convolutional layer is equipped with batch normalization and activation functions to extract and enhance critical features. Finally, an output projection layer maps the integrated features into the pixel space, generating the final reconstructed image.

By leveraging the complementary strengths of local and global features, the feature fusion module significantly enhances the reconstruction accuracy and visual quality of the output image.

### 3.3. Loss Function

SwinTCS is a fully end-to-end approach designed to reconstruct the original image *x* from its measurements *y*. The output y=S(x,A) produced by the sampling module is used as the input to the initial reconstruction module: $I\left(y,\tilde{A}\right)=I\left(S\left(x,A\right),\tilde{A}\right)$. Similarly, the initial reconstruction result serves as the input to the hybrid reconstruction submodule H(·).

To train SwinTCS, the sampling module S(·,A) and the reconstruction module $H(I\left(\cdot ,\tilde{A}\right))$ are jointly optimized. Both the input and the ground truth during training correspond to the original images. The parameters that are learned during the *k*-th stage of the hybrid reconstruction are denoted as $W_{k}$, and the full set of trainable parameters across the *n* stages is represented as $W_{1}∼W_{n}$. To effectively train both the initial and hybrid reconstruction modules from the measurements *y*, SwinTCS uses the mean squared error (MSE) [41] to compute the element-wise discrepancy between the original and reconstructed images. The corresponding loss function is given by

(11) $$L\left(A,\tilde{A},W_{1}∼W_{n}\right):=\frac{1}{2T}\sum_{i=1}^{T}{∥H\left(I\left(S\left(x_{i},A\right),\tilde{A}\right)\right)-x_{i}∥}_{2}^{2}$$

where $x_{i}$ represents the *i*-th training image, and *T* is the total number of training samples.

## 4. Experimental Results

In this section, we analyze the performance of the proposed SwinTCS model through experiments. First, we provide a detailed description of the experimental setup. Next, we conduct comparative experiments between SwinTCS and state-of-the-art methods to assess their robustness under both noise-free and noisy conditions. Afterward, we examine the complexity of SwinTCS, which is crucial for the practical application of the algorithm. Finally, we perform ablation study on the Non-Local Means Denoising module and the AttentionFusion module. It is important to note that these experiments focus on grayscale images (C=1), but our method can be directly applied to multi-channel color images (C=3) on a per-channel basis.

### 4.1. Experimental Settings

#### 4.1.1. Experimental Datasets

For training, we use 400 images from BSD500 dataset for the training set. The validation dataset used is Set 11. All images in the training set are randomly cropped into 200 sub-images, each of size 96×96 pixels, resulting in a total of 100,000 training sub-images. To enhance the diversity of the training data, we apply several image augmentation techniques, including random horizontal flipping, vertical flipping, rotation, and scaling. The testing results are evaluated using four commonly used benchmark datasets: Set5, Set14, BSD100, and Urban100.

#### 4.1.2. Training Details

In our experiments, SwinTCS consists of n=6 iteration stages. The sensing matrix *A* is initialized as a Gaussian matrix, and the initial reconstruction matrix $\tilde{A}$ is initialized as the transpose of the sensing matrix *A*. The block sizes *B* and $B^{\prime }$ are set to 96 and 32, respectively, while the subblock size *P* is set to 8. The learnable iteration step sizes $\lambda_{1}∼\lambda_{n}$ are initialized to 1.0, the weight coefficients $\eta_{1}∼\eta_{n}$ to 0.1, and the trainable shrinkage thresholds $\zeta_{1}∼\zeta_{n}$ to 0.01. The number of attention heads *H* is set to 8. SwinTCS is trained for 200 epochs with a batch size of 64. The learning rate is set to $10^{-3}$ for the first 100 epochs, $10^{-4}$ for epochs 101 to 150, and $10^{-5}$ for the final 50 epochs. The Adam optimizer is used for training. To prevent overfitting and improve generalization, SwinTCS employs a preservation strategy, which is implemented by validating the model after each training epoch. The decision to preserve the trained model is based on the loss of the validation dataset.

### 4.2. Comparisons with State-of-the-Art Methods

We compare SwinTCS with several state-of-the-art compressive sensing models, including GBsR, ReconNet, CSNet, CSformer, ISTA-Net, AMP-Net, TransCS, and OCTUF. Among these, GBsR is a typical traditional compressive sensing algorithm, ISTA-Net and AMP-Net are deep unfolding models that apply traditional mathematical algorithms to deep learning, ReconNet and CSNet are pure deep learning models based on Convolutional Neural Networks (CNNs), and CSformer, TransCS, and OCTUF are compressive sensing models based on Transformers. We evaluate the models using visual quality, PSNR, and SSIM metrics, where higher PSNR and SSIM values indicate better performance.

For fairness, the code for all the comparison models was downloaded from the official websites, and experiments were conducted based on their default settings, using training images from the BSD500 dataset. All experiments were conducted on a platform with the PyTorch 1.9.0 framework, an AMD EPYC 9754 128-core CPU, and a GeForce RTX 4090D GPU. We also compare the models under various sampling rates, specifically τ∈{0.01,0.04,0.10,0.25,0.50}.

#### 4.2.1. Quantitative Comparisons

In this subsection, SwinTCS is compared with other competing methods from objective metrics (PSNR and SSIM). The objective metrics results are calculated for the four datasets (Set5 [42], Set14 [43], BSDS100 [44], and Urban100 [45]) at multiple sampling rates, i.e., τ∈{0.01,0.04,0.10,0.25,0.50}. Based on five sampling rates and four dataset conditions, we conducted comparative experiments between SwinTCS and eight other methods. The experimental results of PSNR and SSIM are shown in Table 1. Optimal results are marked in red and sub-optimal results are marked in blue. The results show that SwinTCS outperforms the other 8 methods at almost all sampling ratios. We perform an averaging operation on the results of the other 8 methods at the same sampling rate for the same dataset so that we can compare them visually. Additionally, we have labeled its increase compared to the average in the SwinTCS column.

Particularly, our model demonstrates outstanding performance on both the Urban100 and Set5 datasets. These two datasets present unique challenges: the Urban100 dataset consists of urban scenes with complex textures and repetitive structures, while the Set5 dataset contains high-resolution images rich in details. The diverse scenes and intricate details of these datasets impose stringent requirements on the feature extraction and detail preservation capabilities of compressive sensing image reconstruction models. The significant improvements in performance can be attributed to our model’s innovative design. By incorporating a Transformer architecture with shifted windowing, it efficiently combines local feature extraction with global dependency modeling. This mechanism overcomes traditional windowing limitations, capturing fine local details and modeling non-local dependencies, particularly enhancing performance on the Urban100 dataset by aggregating relevant features globally and improving image reconstruction. Additionally, the high resolution of Set5 requires precise reconstruction. Our model integrates non-local feature modeling with multi-scale extraction, effectively capturing high-frequency textures while preserving critical details like edges and contours.

At the same time, our model, SwinTCS, outperforms the compared Transformer-based models (TransCS, CSformer, and OCTUF) in terms of performance. SwinTCS consistently exceeds these models in both PSNR and SSIM metrics at the majority of compression rates. At all compression rates, SwinTCS demonstrates significant advantages on the Set5 dataset. On the other three datasets, SwinTCS generally outperforms the three Transformer-based models in our comparative experiments, particularly at high compression rates.

#### 4.2.2. Visual Comparisons

Additionally, we conducted a visual comparison between our model and other competing compressive sensing (CS) methods. Compared to the selected eight models, our approach demonstrates superior performance in recovering finer image details. This is primarily due to the innovative design of our model, which incorporates a Transformer architecture based on shifted windows, enabling it to effectively capture both local features and long-range global dependencies. Notably, our model excels in restoring intricate textures and edge details. The multi-layer feature extraction and cross-window interactions significantly enhance the extraction of fine-grained details.

Furthermore, our model is highly effective in mitigating blocky artifacts, a common challenge in compressive sensing reconstructions at low sampling rates. By integrating cross-window feature interactions and accurate non-local modeling, the model produces smoother and more natural reconstructions. During the deep reconstruction process, our approach also employs multi-scale feature fusion strategies to preserve fine details while effectively suppressing artifacts, resulting in a marked improvement in overall visual quality.

**Figure 4:**
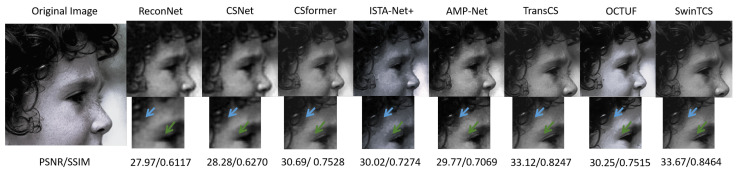
Reconstruction of *Head* image at τ=0.1 by SwinTCS and competing methods. Arrows highlight hair and eyelid.

Figure 4 illustrates the image reconstruction results of the head image from the Set5 dataset at a 10% sampling rate for SwinTCS and other models. From the results, it is evident that SwinTCS outperforms others in recovering image details. For instance, the curly hair, highlighted by the blue arrow, and the nearby fine strands are fully restored only by our model. The double eyelids, indicated by the green arrow, were reconstructed by CSformer, AMP-Net, TransCS, OCTUF, and our model. However, the contours of the eyelids restored by our method are noticeably clearer, especially with finer details.

**Figure 5:**
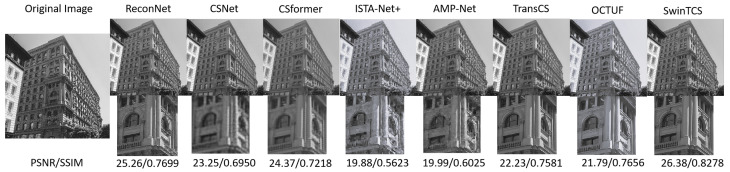
Reconstruction of *Building* image from Urban100 at τ=0.1 by SwinTCS and competing methods.

Figure 5 presents the reconstruction results for building images from the Urban100 dataset at a 10% sampling rate. Our model delivers the highest image quality, restoring detailed features such as walls, windows, and columns with exceptional precision. In comparison, the images reconstructed by ReconNet, CSformer, and CSnet exhibit noticeable blurring, while those by ISTA-Net+ and AMP-Net suffer from significant block artifacts. Overall, our method achieves the best performance, surpassing other models—especially three Transformer-based CS models—in eliminating block artifacts and preserving texture details.

### 4.3. Noise Robustness

We test the robustness of the image reconstruction by adding Gaussian noise to the images to simulate possible noise interference in the channel. The tests are performed on the BSDS100 dataset. The Gaussian noise is set to mean=0, variance σ={0.0005,0.001,0.002,0.003}. Then, the noise robustness of our model (SwinTCS) and four DL-based CS models (ISTA-Net+, CSNet, CSformer, and OCTUF) were compared separately. We use a visual approach for visual comparison under the 0.25 sampling rate and use the metrics PSNR for quantitative analysis at τ∈{0.01,0.04,0.10,0.25}. Optimal results are marked in red and sub-optimal results are marked in blue.

As shown in Table 2, ISTA-Net+ suffers the most from noise interference, followed by OCTUF and CSnet. Notably, CSformer’s performance remains stable as variance increases, with PSNR values fluctuating within 0.01, leaving the table data largely unchanged. SwinTCS outperforms other models except when compared to CSformer at σ=0.003, further validating our NLM module’s effectiveness. However, Gaussian noise still impacts SwinTCS, degrading performance as σ increases.

**Figure 6:**
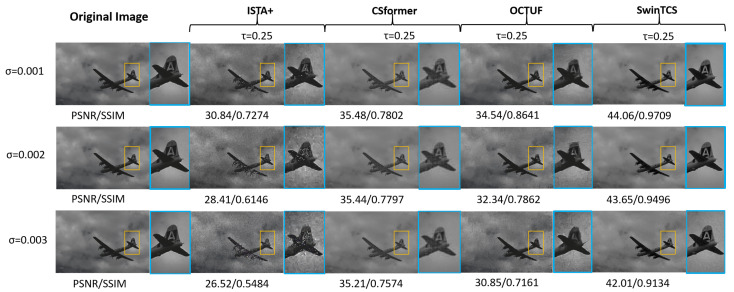
Noise robustness comparison on *airplane* image from BSDS100 at τ=0.25 with Gaussian noise (σ={0.001,0.002,0.003}). Note the recovery of the airplane’s fin.

**Figure 7:**
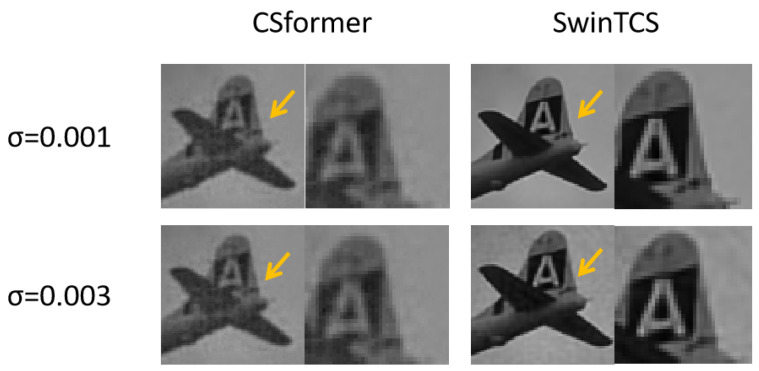
Comparison of CSformer and SwinTCS in Detail Restoration of Airplane Images under Noisy Conditions.

Figure 6 presents the *airplane* image reconstruction at a 25% sampling rate using four models. SwinTCS achieves the highest reconstruction quality, producing clearer images even with noise. ISTA-Net+ shows visible noise in the airplane area, worsening with increased noise. CSformer maintains stable quality but reveals a distinct boundary between the fuselage and sky, along with a visible grid pattern indicating artifacts, as shown in Figure 7. OCTUF reconstructs the fuselage well but struggles with the background, showing unnatural sky and cloud details that worsen with noise. While SwinTCS is also noise-affected, it excels in both background recovery and detailed reconstruction of the airplane tail within the orange box.

### 4.4. Complexity Analysis

We conduct the model complexity analysis of SwinTCS and several competing methods (ReconNet, ISTA-Net+, CSNet, CSformer, AMP-9BM, TransCS, and OCTUF) including the number of giga floating-point operations (GFLOPs) and the number of parameters. GFLOPs are used to measure the time complexity of the model, and the number of parameters is used to measure the spatial complexity of the model. These above metrics are obtained by forward propagating a single 256 × 256 image at a sampling rate of 0.1.

**Figure 8:**
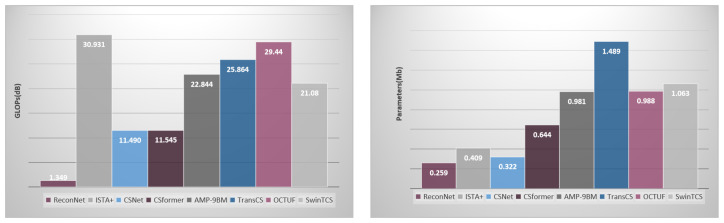
GFLOPs and parameter counts for SwinTCS processing 256 × 256 pixel images with τ=0.1.

As can be seen in Figure 8, ReconNet has very low GFLOPs, mainly due to its single non-iterative model structure. ReconNet is designed for video streaming applications, so some image recovery quality is sacrificed in exchange for faster operation. In addition to this, it can be observed that our model has lower GFLOPs and fewer parameters compared to TransCS and OCTUF, which incorporate a large language model. Additionally, we evaluated the inference time per image on an NVIDIA RTX 4090 GPU. The results show that our model performs competitively compared to other Transformer-based networks. Specifically, on the BSD100 dataset, our model achieves an average reconstruction time of 0.024 s per image, while CSformer requires 0.046 s, TransCS 0.027 s, and OCTUF 0.031 s.

Although there is an exponential increase in each metric compared to CSNet, a comparison of the image recovery quality in Table 1 indicates that it is worth sacrificing model complexity to some extent.

Ultimately, there is a trade-off between the quality of reconstructed images and model complexity. SwinTCS has been successful in improving image quality compared to previous models without a significant increase in model complexity, achieving lower GFLOPs and fewer parameters than some models (e.g., AMP-9BM, TransCS, and OCTUF).

### 4.5. Ablation Experiments

#### 4.5.1. Non-Local Means Denoising

**Figure 9:**
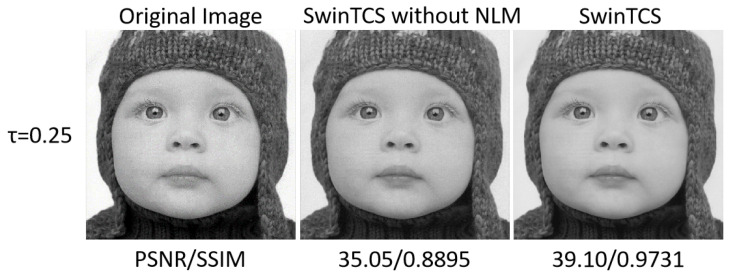
Comparison of visualizations with and without NLM at different sampling rates τ=0.25.

To evaluate the effectiveness of Non-Local Means Denoising, we conducted ablation studies on the BSDS100 dataset. Specifically, we compared SwinTCS with and without Non-Local Means Denoising. The results, as illustrated in Table 3 and Figure 9, show that incorporating Non-Local Means Denoising enhances the quality of the reconstructed images. This improvement is particularly significant at higher sampling rates. The reason is likely that Non-Local Means Denoising acts as a smoothing component within the model. It helps to produce more natural and realistic reconstructed images, which are closer to the original images. Consequently, this mechanism improves the overall stability of the deep learning model during the image reconstruction process.

To further verify the effectiveness of Non-Local Means Denoising, ablation experiments were also conducted on the Set5 dataset. As shown in Table 3 and Figure 9, the incorporation of Non-Local Means Denoising significantly enhances the quality of reconstructed images, especially at high sampling rates. In Figure 9, the parrot image reconstructed without the Non-Local Means (NLM) module exhibits noticeable blocky artifacts. In contrast, the parrot image reconstructed with the NLM module not only effectively eliminates these blocky artifacts but also shows superior performance in detail texture representation. This improvement is likely due to the smoothing effect of Non-Local Means Denoising, which makes the reconstructed images more natural and closer to the original images. Consequently, this mechanism contributes to the overall stability of the deep learning model during the image reconstruction process.

#### 4.5.2. Attention Fusion

**Figure 10:**
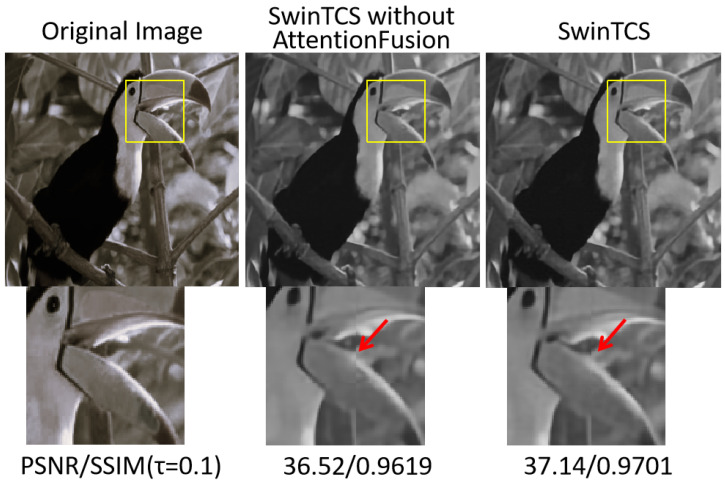
Comparison of visualizations with and without AttentionFusion at different sampling rates τ=0.1.

To evaluate the impact of AttentionFusion, we conducted ablation experiments on the Set5 dataset. Specifically, we compared the reconstruction performance of SwinTCS with and without AttentionFusion, where the latter simply concatenates the features from the CNN and Transformer branches. As illustrated in Figure 10, incorporating AttentionFusion significantly improves the quality of reconstructed images. This enhancement is primarily attributed to AttentionFusion’s ability to adaptively aggregate spatial and contextual features, thereby facilitating a more effective information flow across different regions of the image. By strengthening the connections among key features, the reconstructed images generated by AttentionFusion exhibit greater visual coherence and higher perceptual quality. Moreover, this mechanism enhances the model’s stability and generalization in complex image reconstruction tasks.

As shown in Figure 10, in the absence of AttentionFusion, the reconstructed parrot image exhibits noticeable inconsistencies in texture details along with certain spatial distortions, with particularly obvious blocky artifacts around the beak. In contrast, when AttentionFusion is introduced, the local features of the reconstructed image are effectively refined, resulting in clearer texture details and better preservation of overall structural integrity. This improvement is likely due to AttentionFusion leveraging attention mechanisms to guide feature aggregation, selectively enhancing critical region information while suppressing redundant information. Consequently, AttentionFusion not only improves the perceptual realism of reconstructed images but also enhances the robustness of the deep learning model in the image reconstruction process.

## 5. Conclusions and Future Work

This paper proposes a Transformer-based compressive sensing framework, SwinTCS, which integrates the shifted window attention mechanism from the Swin Transformer and the local feature extraction capabilities of CNNs. The framework effectively addresses boundary artifacts and noise sensitivity in traditional compressive sensing models. By leveraging a feature fusion mechanism, SwinTCS achieves a synergistic optimization of global and local information, significantly improving reconstruction quality.

Additionally, SwinTCS incorporates a noise suppression module based on Non-Local Feature Means (NLM), enhancing adaptability to diverse noise conditions. The experimental results demonstrate that SwinTCS outperforms existing deep learning methods at various compression ratios with lower computational complexity and superior reconstruction performance.

While our model demonstrates strong performance, several practical challenges remain when deploying it in real IoT environments. These include limited memory, hardware constraints, fluctuating data rates, and high sensor noise, which can affect real-time performance. Furthermore, processing visual data may raise privacy concerns in sensitive applications such as healthcare or surveillance. Ethical issues, such as the potential for misuse or unintended reconstruction of private content, also require attention. These factors highlight the importance of responsible deployment and robust system design. In terms of practical application, SwinTCS is suitable for various scenarios including smart healthcare, transportation systems, and home security, where efficient and reliable image reconstruction is crucial under constrained resources.

In future work, we plan to address the following directions:

- Optimize the shift strategy and network depth to enhance performance on complex visual scenes and better balance global–local information extraction.
- Dynamically adjust learning rates and optimization strategies to improve convergence stability and training efficiency.
- Further accelerate the model to meet the low-latency requirements of real-time IoT applications, considering deployment on edge devices with strict resource limitations.
- Investigate privacy-preserving mechanisms such as encryption or federated learning to protect sensitive visual data during reconstruction.
- Develop ethical guidelines for responsible deployment and evaluate the social impact of image reconstruction in practical applications.

## Figures and Tables

**Table 1:** PSNR and SSIM comparisons for MambaCS and 8 baselines on Set5, Set14, BSDS100, Urban100 datasets at sampling rates of 0.01, 0.04, 0.1, 0.25, and 0.5.

Datasets	Method	GBsR		ReconNet		CSNet		Csformer		ISTA-Net+		AMP-Net		TransCS		OCTUF		Average		SwinTCS	
		(TIP2014)		(CVPR2016)		(TIP2019)		(TIP2023)		(CVPR2018)		(TIP2021)		(TIP2022)		(CVPR2023)				(Ours)	
	Ratio	PSNR	SSIM	PSNR	SSIM	PSNR	SSIM	PSNR	SSIM	PSNR	SSIM	PSNR	SSIM	PSNR	SSIM	PSNR	SSIM	PSNR	SSIM	PSNR	SSIM
Set5	0.01	18.89	0.4919	22.34	0.5895	22.74	0.5864	23.90	0.6571	20.25	0.5608	20.43	0.5776	21.29	0.5787	——	——	21.41	0.5774	23.97(2.56↑)	0.6311(0.0537↑)
	0.04	23.82	0.7032	24.89	0.7392	25.22	0.7307	27.70	0.7931	24.65	0.7219	24.06	0.7274	25.19	0.7378	——	——	25.08	0.7362	28.97(3.89↑)	0.8395(0.1033↑)
	0.1	27.12	0.8401	27.89	0.8418	27.92	0.8413	30.36	0.8676	29.16	0.8515	29.56	0.8682	29.77	0.8805	30.39	0.8751	29.02	0.8583	33.73(4.72↑)	0.9328(0.0745↑)
	0.25	31.72	0.8832	31.9	0.8993	33.07	0.9160	33.36	0.9230	34.17	0.9372	34.88	0.9486	35.15	0.9510	35.25	0.9300	33.69	0.9235	38.89(5.20↑)	0.9697(0.0462↑)
	0.5	37.56	0.9599	37.94	0.9611	38.28	0.9623	39.11	0.9639	39.49	0.9806	40.50	0.9868	41.31	0.9909	40.63	0.9744	39.35	0.9724	43.64(4.29↑)	0.9895(0.0170↑)
Set14	0.01	18.26	0.4884	21.73	0.5583	21.92	0.5485	22.73	0.5681	19.82	0.5414	20.02	0.5430	21.03	0.5454	——	——	20.79	0.5419	21.97(1.18↑)	0.5481(0.0062↑)
	0.04	22.69	0.6534	24.25	0.6755	24.72	0.7062	25.54	0.6922	23.78	0.6808	23.53	0.6880	24.78	0.6958	——	——	24.18	0.6845	25.59(1.41↑)	0.7119(0.0279↑)
	0.1	26.04	0.7836	27.15	0.8002	27.45	0.8037	27.71	0.7886	28.26	0.8289	28.76	0.8354	28.97	0.8552	27.55	0.7827	27.74	0.8098	28.79(1.05↑)	0.8381(0.0213↑)
	0.25	30.82	0.8938	31.2	0.8357	31.98	0.8959	31.35	0.8956	32.92	0.9386	33.38	0.9412	33.75	0.9464	31.4	0.8937	32.10	0.9051	33.91(1.81↑)	0.9342(0.0291↑)
	0.5	35.47	0.9119	35.86	0.9210	36.39	0.9572	37.07	0.9569	38.28	0.9685	38.52	0.9722	39.42	0.9757	36.12	0.951	37.14	0.9518	39.57(2.43↑)	0.9725(0.0207↑)
BSD100	0.01	20.14	0.5030	22.96	0.5843	23.21	0.5884	23.61	0.5954	21.86	0.5674	21.97	0.5723	22.44	0.5856	——	——	22.31	0.5709	23.11(0.80↑)	0.5791(0.0082↑)
	0.04	24.12	0.6919	25.58	0.7441	25.87	0.7432	26.58	0.7406	25.23	0.7223	25.12	0.7289	26.25	0.7393	——	——	25.54	0.7300	25.92(0.38↑)	0.7305(0.0005↑)
	0.1	27.88	0.8230	28.12	0.8315	28.31	0.8381	29.92	0.8314	30.04	0.8443	30.24	0.8666	30.79	0.8761	26.32	0.738	28.95	0.8311	30.52(1.57↑)	0.8434(0.0123↑)
	0.25	32.21	0.8688	32.26	0.8872	33.70	0.9093	34.75	0.9085	35.04	0.9295	35.45	0.9352	36.16	0.9498	29.83	0.8675	33.68	0.9070	35.46(1.78↑)	0.9269(0.0199↑)
	0.5	37.63	0.9487	38.04	0.9540	38.49	0.9579	38.85	0.9756	40.73	0.9773	41.34	0.9745	42.07	0.9888	34.58	0.9481	38.97	0.9656	40.06(1.09↑)	0.9714(0.0058↑)
Urban100	0.01	18.26	0.3786	18.02	0.3785	17.74	0.3724	20.85	0.4938	16.66	0.3731	17.00	0.3563	18.02	0.3711	——	——	18.08	0.3919	19.69(1.61↑)	0.4943(0.1024↑)
	0.04	21.69	0.5258	21.72	0.6688	20.79	0.5781	22.97	0.6533	19.66	0.5369	19.93	0.5415	23.23	0.7107	——	——	21.43	0.6021	22.99(1.57↑)	0.6959(0.0973↑)
	0.1	25.04	0.7385	26.35	0.7937	25.54	0.7525	25.06	0.7558	23.51	0.7199	23.11	0.6946	26.72	0.841	26.45	0.8156	25.22	0.7639	26.49(1.27↑)	0.8279(0.0639↑)
	0.25	28.28	0.8218	30.08	0.8589	27.80	0.8039	27.51	0.8497	28.9	0.8831	28.37	0.8672	31.7	0.9329	31.16	0.9224	29.22	0.8674	31.85(2.63↑)	0.9345(0.0670↑)
	0.5	31.47	0.9011	33.79	0.9257	30.36	0.9394	30.06	0.9096	34.35	0.9569	34.27	0.9529	37.18	0.976	36.02	0.967	33.44	0.9411	38.24(4.80↑)	0.9781(0.0370↑)

**Table 2:** PSNR and SSIM comparisons for SwinTCS and other baselines on BSDS100 datasets at different sampling rates and different noise levels σ.

σ	SR (τ)	ISTA-Net+	CSformer	CSNet	OCTUF	SwinTCS
		PSNR / SSIM				
0.0005	0.01	18.96/0.3387	17.37/0.4654	20.77/0.4449	—	22.45/0.4581
	0.04	21.43/0.4589	19.51/0.5534	22.78/0.5244	—	25.09/0.6997
	0.1	23.72/0.5912	22.67/0.6410	23.43/0.5622	25.60/0.7012	27.33/0.7909
	0.25	26.77/0.7559	25.69/0.7512	24.60/0.6343	28.10/0.8021	31.07/0.8828
0.001	0.01	18.61/0.3020	17.37/0.4654	20.76/0.4444	—	22.01/0.3953
	0.04	20.88/0.4155	19.51/0.5534	22.76/0.5228	—	24.15/0.5569
	0.1	22.92/0.5418	22.67/0.6409	23.40/0.5599	25.12/0.6783	26.12/0.6897
	0.25	25.55/0.7067	25.68/0.7508	24.55/0.6296	27.13/0.7531	28.80/0.7873
0.002	0.01	18.14/0.2546	17.37/0.4654	20.74/0.4436	—	21.60/0.3104
	0.04	20.12/0.3577	19.50/0.5534	22.70/0.5197	—	23.96/0.4715
	0.1	21.90/0.4779	22.67/0.5558	23.34/0.5599	24.40/0.6287	25.36/0.5836
	0.25	24.08/0.6456	25.67/0.7504	24.42/0.6206	25.83/0.6801	26.32/0.6706
0.003	0.01	17.81/0.2223	17.37/0.4654	20.73/0.4429	—	21.12/0.2745
	0.04	19.58/0.3189	19.50/0.5534	22.65/0.5168	—	22.67/0.4194
	0.1	21.19/0.4356	22.67/0.6408	23.27/0.5519	23.85/0.5921	24.06/0.6006
	0.25	23.09/0.6051	25.66/0.7500	24.30/0.6123	24.91/0.6265	25.75/0.7306

**Table 3:** Experiments for the validity of Non-Local Means Denoising module.

	SR(τ)	0.01	0.04	0.1	0.25	0.5
SwinTCS (PSNR/SSIM)	−NLM	22.12/0.4988	25.95/0.7551	31.92/0.8529	36.67/0.9138	42.19/0.9616
	+NLM	23.97/0.6311	28.97/0.8695	33.73/0.9328	38.89/0.9697	43.64/0.9895

## Data Availability

The original contributions presented in the study are included in the article; further inquiries can be directed to the corresponding author.
